# Protocol for Development of an Evidence-Based Rapid Readiness Screen

**DOI:** 10.1227/neuprac.0000000000000225

**Published:** 2026-04-17

**Authors:** Simon Oczkowski, Thomas Bayuk, John Basmaji, Bradley A. Dengler, Michael McCrea, Sameer Sharif, Katie Stout, Gregory W. J. Hawryluk, Halinder S. Mangat, Jamshid Ghajar

**Affiliations:** 1 Department of Medicine, Division of Critical Care, McMaster University, Hamilton, Ontario, Canada;; 2 Department of Health Research Methods, Evidence, and Impact, McMaster University, Hamilton, Ontario, Canada;; 3 Guidelines in Intensive Care, Development, and Evaluation (GUIDE) Group, Hamilton, Ontario, Canada;; 4 Department of Neurology, Uniformed Services University of the Health Sciences, Bethesda, Maryland, USA;; 5 Division of Critical Care, Department of Medicine, London Health Sciences Center, London, Ontario, Canada;; 6 Departments of Surgery and Neurology, Uniformed Services University of the Health Sciences (USU), Bethesda, Maryland, USA;; 7 Department of Neurosurgery, Walter Reed National Military Medical Center, Bethesda, Maryland, USA;; 8 Military Traumatic Brain Injury Initiative (MTBI^2^), Bethesda, Maryland, USA;; 9 Department of Neurosurgery, Medical College of Wisconsin, Milwaukee, Wisconsin, USA;; 10 Department of Medicine, Division of Emergency Medicine, McMaster University, Hamilton, Ontario, Canada;; 11 Traumatic Brain Injury Center of Excellence, Defense Health Agency, Silver Spring, Maryland, USA;; 12 Cleveland Clinic Lerner College of Medicine, Case Western Reserve University School of Medicine, Cleveland, Ohio, USA;; 13 Brain Trauma Foundation, Palo Alto, California, USA;; 14 Kansas University Medical Center Research Institute, Kansas City, Kansas, USA

**Keywords:** Assessment, Attention, Concussion, Intoxication, Military, Screen, Screening, Sleep, Sport

## Abstract

In combat and sport, there is an urgent need to identify readiness for performance to decide on removal from and return to activity. Cognitive readiness, including attention, can be impaired for many potentially coexisting reasons, including concussive or subconcussive injury, insufficient sleep/fatigue, and intoxication. Standard tests of concussion, sleep deprivation, and intoxication are often lengthy or require specialized skill or equipment to conduct. The ideal readiness screen should predict readiness or be associated with surrogate measures of readiness, be sensitive to multiple conditions that may impair readiness, and be reliable and rapid. This study outlines the methodology for an evidence-based process to develop multimodal rapid screening tests for readiness that are applicable across a broad range of environments, including military far-forward/sports sideline, garrison, and clinic.

**ABBREVIATIONS:**
COI: conflict of interest
DoW: Department of War
EtD: evidence-to-decision
GRADE: grading of recommendations assessment development and evaluation
MACE 2: Military Acute Concussion Evaluation 2
PICO: population intervention comparator outcome
TBI: traumatic brain injury.

A top priority for the Department of War (DoW) is to optimize the cognitive and physical capabilities of warfighters to ensure individual and unit readiness to deploy, and equally importantly, to return to engagement following any injury. The DoW Warfighter Brain Health Initiative supports research to monitor and optimize warfighters' brain function in response to the cognitive and physical demands warfighters face, such as sleep loss, head injury, environmental hazards, and emotional stress. Ensuring that warfighters maintain peak cognitive performance is vital for mission success.

Cognitive readiness can be defined as an optimal brain state enabling the precise coordination of sensory inputs and motor responses that are appropriate to the task at hand.^1^ However, current measures of attention do not adequately assess warfighters' ability to function continuously in the demanding environments they face. Identifying evidence-based specific features of cognitive readiness and developing a cognitive readiness screen are essential steps in establishing strategies for sustaining warfighter performance.

Despite DoW's ongoing research efforts, the mechanisms underlying cognitive readiness in warfighters remain poorly understood; furthermore, the high-stress conditions of combat pose significant challenges for assessing readiness in real-world operational contexts. A 2017 report, “Military Cognitive Performance and Readiness Assessment Initiative,” highlighted the absence of objective assessments to predict performance or injury risk.^2^ More objective indicators, such as changes in speech, eye movement, and balance, are essential in gauging cognitive readiness. DoW recognizes the need for better measures, particularly concerning concussion subtypes and associated conditions like sleep/fatigue, to inform rapid decisions for removal and return to duty. Other requirements for a readiness test are speed of administration, option for self-administration, and flexibility in setting thresholds that are appropriate for the task at hand. The current cognitive assessments for sports and military are the Sport Concussion Assessment Tool for Adolescents (13 years +) and Adults,^3^ the Military Acute Concussion Evaluation 2 (MACE 2),^4^ and the Automated Neuropsychological Assessment Metrics,^5^ each of which takes from 10 to 30 minutes to administer. Environments such as far-forward austere military settings, sports sidelines, and garrison and training require rapid screening, ideally less than 1 minute in a far-forward military setting. In addition, the thresholds for a screen failure will be different for special forces, garrison surveillance, and sports sidelines and may compare variously to baselines, group norm, or age-normative results.

The “Progressive Return to Activity Algorithm” from the traumatic brain injury (TBI) center of excellence has a detailed evaluation for TBI assessment and return.^6^ However, it has important limitations. First, the current standard evaluation tool, the MACE 2, is impractical in settings requiring quick decisions because of its testing and time requirements.^7^ Second, the current algorithm emphasizes concussive head injury, and overlooks other important conditions which may impair readiness in the military setting, such as blast overpressure injuries as a result of explosion pressure waves, either singly or repetitively. The DoW's recent memorandum, “Department of Defense requirements for Managing Brain Health Risks from Blast Overpressure,” identified potential adverse effects on brain health and cognitive performance from exposure to blast overpressure, both acute and chronic.^8^ Because they lack a single injury event, impairments from overpressure (subconcussive injury) may be overlooked when assessing readiness. Beyond head injuries, other conditions (sleep/fatigue, intoxication) may coexist and may result in dynamic impairments that inhibit individuals' readiness. Thus, the current paradigm of concussion diagnosis leaves a gap in the initial, very early, assessment of readiness in situations where multiple and dynamic impairments may exist, resources are limited, and the environment is not suitable for a detailed cognitive evaluation (Figure).

**FIGURE.**
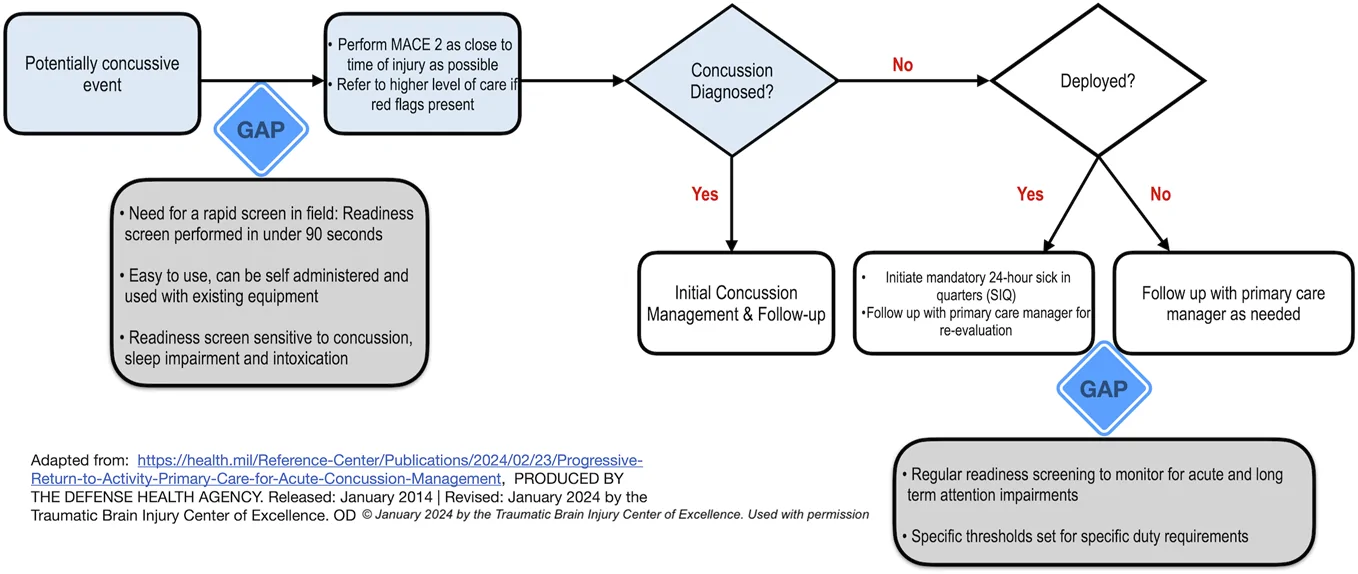
Initial assessment and management of potentially concussive events and the gaps requiring a rapid readiness screen. Adapted from: https://health.mil/Reference-Center/Publications/2024/02/23/Progressive-Return-to-Activity-Primary-Care-for-Acute-Concussion-Management, Produced by the Defense Health Agency. Released: January 2014. Revised: January 2024 by the TBICoE. OD*.* © *January 2024 by the TBICoE. Used with permission.*

A readiness screen in these situations could establish and monitor normative readiness thresholds and need to be passed to resume activity. This is a paradigm evolution, wherein screening/diagnosis of concussion is transitioned to assessment of cognitive readiness for activity. In dynamic environments, whether combat or sports, the diagnosis of concussion, is less meaningful than the determination of cognitive readiness, a term encompassing more than just concussive or subconcussive injury but also sleep deprivation, intoxication, or any other condition that impairs readiness or attention.

The Brain Trauma Foundation's past concussion guidelines have progressed through 2 key steps. The 2014 “Concussion Guidelines Step 1” identified attention and balance impairments as prevalent within the first 24 hours following a concussive event.^9^ In 2019, “Concussion Guidelines Step 2” provided evidence for 5 concussion subtypes (cognitive, ocular-motor, headache/migraine, vestibular, and anxiety/mood), along with 2 associated conditions (cervical strain and sleep disturbance).^10^

This project will address DoW's lack of a cognitive readiness screen that is usable in environments where administering the MACE 2 is not feasible. The proposed rapid readiness screen focuses on eye, verbal, and motor subtypes, which aligns it with the Glasgow Coma Scale categories of evaluation for TBI. The rapid readiness screen will meet the following criteria.

- Usable in garrison, training, or austere environments
- Usable with minimal training
- Noninvasive and rapid (seconds to a couple of minutes)
- Highly reliable
- Robust for harsh conditions, using minimal equipment

## METHODS

### Working Group Membership

Brain Trauma Foundation's leadership assembled a steering committee to provide project governance. The steering committee identified key stakeholders and subject matter experts familiar with a variety of contexts (military, sports) and conditions (concussion, sleep deprivation, intoxication), including a patient representative. The Guidelines in Intensive Care Medicine, Development, and Evaluation Group, which brings expertise in the use of grading of recommendations assessment, development and evaluation (GRADE) methodology for guideline development, will provide methodological support.^11^

### Conflict of Interest Identification and Management

All panelists will be required to disclose any potential conflict of interest (COI) in advance of participating in the project. Significant COIs may include financial (any financial relationships with entities related to a question) or intellectual (being a grant holder or senior investigator on any studies included in an evidence review for a given question). To allow expert input on evidence interpretation, panelists with a COI may participate in reviewing and interpreting the evidence but will be recused from participating in discussion of the recommendation and its wording.

### Question Selection

The steering committee developed a list of eye, verbal, and motor tests as potential candidates for inclusion in rapid readiness screens. In addition, a question addressing the use of thresholds based on group means vs individual baseline measurements was included. These 13 questions were structured in the population, intervention, comparator, outcome (PICO) format (Table 1).

**TABLE 1.** PICO Questions and Inclusion/Exclusion Criteria

PICO question	Population	Screening intervention/test	Outcomes	Study designs	Exclusion
1. Eye subtypes					
1. Convergence/binocular eye movement or eye fixation tests	Patients age >16 years with potentially impaired cognition/attention or function because of one or more of (1) mild traumatic brain injury or potential concussion/subconcussive head injury (eg, sports, combat, trauma) (2) sleep deprivation, or (3) intoxication (alcohol or cannabis) within 72 h of injury/insult	Binocular convergence test (binocular coordination and vergence) or eye fixation test, any test format	Prediction (sens/spec) or association with readiness to return or surrogate test of readiness, attention, or cognition; responsiveness to target condition	Diagnostic accuracy studies of the test; case-control studies; or randomized controlled trials comparing screening/diagnostic strategies of the test	Studies published in abstract form without extractable data; studies not published in English; studies published before 2000; studies in children (age <16 years)
2. Saccadic eye movement tests		Saccadic eye movement tests, any test format			
3. Smooth pursuit eye movement tests		Smooth pursuit eye movement tests, any test format			
4. Pupil reaction tests		Pupil reaction tests (including quantitative pupillometry)			
5. Vestibulo-ocular reflex tests or fixation tests		Vestibulo-ocular reflex testing or fixation test, any test format			
6. Visual motion sensitivity tests		Visual motion sensitivity tests, any test format			
2. Verbal subtypes					
7. Voice biomarker tests	Patients age >16 years with potentially impaired cognition/attention or function because of 1 or more of (1) mild traumatic brain injury or potential concussion/subconcussive head injury (eg, sports, combat, trauma) ( 2) sleep deprivation, or (3) intoxication (alcohol or cannabis) within 72 h of injury/insult	Voice biomarker tests, any test format	Prediction (sens/spec) or association with readiness to return or surrogate test of readiness, attention, or cognition; responsive ness to target condition	Diagnostic accuracy studies of the test; case-control studies; or randomized controlled trials comparing screening/diagnostic strategies of the test	Studies published in abstract form without extractable data; studies not published in English; studies published before 2000; studies in children (age <16 years)
8. Verbal working memory tests		Verbal working memory tests, any test format			
9. Vestibular oculomotor screening test		Vestibular oculomotor symptoms tests, any test format			
10. Mood disorders screening		Mood disorder screening test for depression, anxiety, or post-traumatic stress disorder, any test format			
3. Motor subtypes					
11. Static balance tests	Patients age >16 years with potentially impaired cognition/attention or function because of 1 or more of (1) mild traumatic brain injury or potential concussion/subconcussive head injury (eg, sports, combat, trauma) ( 2) sleep deprivation, or (3) intoxication (alcohol or cannabis) within 72 h of injury/insult	Static balance tests, any test format	Prediction (sens/spec) or association with readiness to return or surrogate test of readiness, attention, or cognition; responsive ness to target condition	Diagnostic accuracy studies of the test; case-control studies; or randomized controlled trials comparing screening/diagnostic strategies of the test	Studies published in abstract form without extractable data; studies not published in English; studies published before 2000; studies in children (age <16 years)
12. Tandem and dynamic gait tests		Tandem and/or dynamic gait tests, any test format			
4. Thresholds					
13. Thresholds for removal and return	Patients age >16 years with potentially impaired cognition/attention or function due to 1 or more of (1) mild traumatic brain injury or potential concussion/subconcussive head injury (eg, sports, combat, trauma) (2) sleep deprivation, or (3) intoxication (alcohol or cannabis) within 72 h of injury/insult	Test cutoff based upon individual patient baseline testing vs population means vs normative means vs best medical judgment	Test cutoff based upon alternative threshold	Diagnostic accuracy studies of the test; case-control studies; or randomized controlled trials comparing screening/diagnostic strategies of the test	Studies published in abstract form without extractable data; studies not published in English; studies published before 2000; studies in children (age <16 years)

PICO, population, intervention, comparator, outcome.

### Definition and Classification of Outcomes

Following GRADE guidance, the steering committee prioritized outcomes as “critical,” “important,” or “unimportant” for decision-making.^12^ Three outcomes were identified as being “critical” to considering inclusion of a test within a rapid readiness screen.

1. The ability of the test to identify an individual's readiness to return to function. As there is no “gold standard” for assessment of readiness, we anticipate several ways this would be inferred from the published literature. This could be assessed with direct evidence (demonstration of adequate function and performance in the field) or indirect evidence (test correlation with either or both of the following: (1) clinician judgment of readiness to return; (2) measures of readiness and attention, including simulated tasks, cognitive testing, physical testing, and/or persistent symptoms). Any such outcome measure reported in the study will be used in the evidence summary.
2. Responsiveness of the test to the underlying condition (ie, changes in the test are correlated with changes in condition [concussion, sleep deprivation, and intoxication]).
3. Reliability of the test (ie, test-retest reliability).

### Literature Review

To inform the development of a rapid readiness screen, we will conduct systematic reviews for each PICO question, based on key terms and previously published systematic reviews. With the assistance of a medical librarian, we will search multiple databases, including Medline, Embase, and Cochrane Central, for studies from 2000 to present, recognizing that older publications may not reflect current technology or contemporary practice.

### Inclusion Process for Articles

The results of the database searches will be uploaded to COVIDENCE (Covidence systematic review software 2025, Veritas Health Innovation, https://www.covidence.org) for database management and screening. Detailed inclusion/exclusion criteria for each PICO are provided in Table 1. We will include studies with >50% patients aged 16 years or older and that screened for early impairment (ideally within 72 hours of event, [ie, concussion, subconcussive/blast overpressure, intoxication, or sleep deprivation]). Articles published as abstracts without sufficient data to allow for data extraction, or not in English, will be excluded. Articles will be screened independently for inclusion by 2 reviewers as titles and abstracts and at a secondary full-text review stage. Titles and abstracts included by either reviewer will advance to full-text screening, with adjudication by a third reviewer if a consensus on inclusion/exclusion cannot be reached.

### Process of Information Extraction and Evidence Synthesis

Data extraction will be performed by a member of the methodology team with secondary verification by another methodology team member. Data extraction will include key study characteristics, study author, and year; age of participants; population characteristics; screening test details; platform used; time required for test; comparison test; description of how thresholds/cutoffs were developed; key study results; and characteristics relevant to assessment of study risk of bias.

If feasible, meta-analysis will be conducted by pooling diagnostic test accuracy results, including sensitivity and specificity, to calculate receiver operating curve and associated area under the curve; positive and negative likelihood ratios; and positive and negative predictive values, with associated 95% CIs. We will calculate pooled estimates using both fixed-effects and random-effects models, considering the risk of small-studies effects when only a small number of trials provided data for a given outcome.^13^ When between-study differences in reported outcomes prohibit data pooling, data will be summarized narratively.

### Assessment of the Evidence

Risk of bias for diagnostic test accuracy will be assessed using the Quality Assessment of Diagnostic Accuracy Studies-2 instrument; we anticipate that many papers are likely to be at a high risk of bias because of the use of case-control designs.^14^ The methodology team will use a modified GRADE approach to assess the certainty of evidence for each outcome, as each PICO is considering a screening test for inclusion within a multimodal screening algorithm, rather than as a test for direct clinical application.^15^ Following GRADE guidance, we will assess the certainty of evidence, rating it as high, moderate, low, or very low, according to study risk of bias, inconsistency, indirectness, imprecision of estimates, and risk of publication bias in the body of evidence.^16-20^ In the context of diagnostic tests, observational studies of diagnostic test accuracy start as “high” certainty evidence and are rated down if most included studies are considered to be at “high” risk of bias; if studies demonstrate tests as having inconsistent ability to predict readiness; if the evidence is indirect because of the population, test used, timing of test, or outcome measurements; or if the available studies provide only imprecise estimates of test accuracy.

A priori we determined that sensitive tests of readiness would be prioritized over specific, with false negatives of 1 to 2 per 100 identified as a “small” amount of imprecision, 3 to 5 per 100 as a “moderate” amount of imprecision, 6-9 per 100 as a “large” amount of imprecision, and 10 or more per 100 as a “very large” amount of imprecision. Corresponding false-positive rates would be higher (<10 identified as “small,” 10-15 per 100 as “moderate,” 16-20 as “large,” and more than 20 per 100 as “very large” degrees of imprecision), reflecting the emphasis on identifying individuals who are unready. These relative thresholds were determined by consensus within the leadership team based upon the anticipated higher risk of returning “unready” individuals to active combat or sport, vs the lower risk of withholding “ready” individuals from activity. The exact threshold cutoffs were chosen as ranges within the expected values and clinical relevance of sensitivity and specificity for each test in military and athletic settings. We anticipate that several PICOs may lack quantitative data suitable for meta-analysis and thus will be summarized as narrative evidence.^21^

When summarizing the effects, we will follow GRADE's recommendations for informative wording to describe the effect estimates.^22^ The ratings of the evidence with their meaning and interpretation are presented in Table 2.

**TABLE 2.** Grade of Levels of Evidence and Their Interpretation

Certainty of evidence	Interpretation
High	There is great confidence that the true effect is close to the estimate; we have high certainty of the test's ability to predict readiness.
Moderate	There is moderate confidence in the estimate of the effect; it is likely that the test can predict readiness, but the possibility exists that it may not.
Low	Confidence in the estimate of the effect is limited; although it is possible that the test can predict readiness, the evidence is substantially uncertain.
Very low	One can have very little confidence in the estimate of the effect; we have little idea of the test's ability to predict readiness.

### Formulation of Recommendations

The working group will use a modified GRADE evidence-to-decision (EtD) framework to determine the direction and strength of each recommendation.^23^ In contrast to a standard diagnostic test EtD, which considers first a test's diagnostic sensitivity/specificity and second the clinical implications, the modified EtD will consider each test's ability to contribute to a rapid readiness screen by assessing the test's desirable and undesirable characteristics, trade-offs, resource implications, acceptability, and feasibility. Each recommendation will include a strength (strong vs conditional) and directionality (for or against using the test). The lower the certainty of the evidence, or the more closely balanced the desirable and undesirable elements of the test, the more likely a conditional, rather than strong, recommendation will be made. A subset of working group members will be assigned to each PICO to review the evidence and develop a draft recommendation.

### Consensus Statements and Algorithm Development

The draft recommendations were presented at an in-person meeting in Bethesda, MD, in May 2025. The panel will review the draft recommendations developed by each PICO working group. In addition to the formal GRADE evidence-based recommendations, the panel may develop additional expert consensus statements to provide clinical context and facilitate implementation. These consensus statements will be clearly identified as such to avoid conflation with the formal evidence-based recommendations. The consensus statements will require an 80% vote of approval by panelists without a relevant COI.

The panel will use the approved recommendations to guide the development of a rapid readiness screening algorithm in 3 potential environments, each with different resource constraints and practical considerations (Table 3). Each readiness screening test with a conditional or strong recommendation will be considered for inclusion in the readiness algorithms. Screening tests with a conditional recommendation may or may not be included in the final algorithms after considering the screening test's ability to predict readiness, duration of test, strength of evidence, costs, device platform, and feasibility. Tests with a strong recommendation will be included unless there is a highly compelling reason for their exclusion. The algorithms will require 80% agreement of participants without relevant COI for final approval.

**TABLE 3.** Potential Environments for a Rapid Readiness Screen

Environment	Characteristics
Austere	Description: military: far forward; sports: sidelines; civilian: field Time: under 60 s Platforms: phone, tablet, none Sensitivity/specificity: high sensitivity, low specificity Reliability: high Comparator: military: baseline/group; sports: baseline/group; civilian: norms Threshold: military: high; sports: high; civilian: activity dependent
Garrison	Description: military: barracks/training; sports: indoors; civilian: office Time: under 90 s Platforms: phone, tablet, none Sensitivity/specificity: medium sensitivity, medium specificity Reliability: high Comparator: military: baseline/group; sports: baseline/group; civilian: norms Threshold: military: medium; sports: medium; civilian: activity dependent
Clinic	Description: military: clinic; sports: sports clinic; civilian: medical clinic Time: under 10 min Platforms: Any device Sensitivity/specificity: medium sensitivity, high specificity Reliability: medium Comparator: military: baseline/diagnostic; sports: baseline/diagnostic; civilian: baseline/diagnostic Threshold: military: medium; sports: medium; civilian: activity dependent

### Incorporation of Artificial intelligence

Artificial intelligence will be used to facilitate the writing of the study, but all aspects of the project will be overseen, reviewed, and edited by human panel members. Details of the artificial intelligence software will be provided as a supplement in the final manuscript.

## CONCLUSION

This project will use modified GRADE methodology to provide a rigorous literature review and consensus process to develop algorithms for a rapid readiness screen for use across multiple environments.
